# Supplementary material for: Conditional chemoconnectomics (cCCTomics) as a strategy for efficient and conditional targeting of chemical transmission
Source: eLife. 2024 Apr 30;12:RP91927. doi: 10.7554/eLife.91927 (PMC11060718; doi:10.7554/eLife.91927)
Supplement: Figure 3—source data 3. [file elife-91927-fig3-data3.zip › Figure 3 source data3 part 1.zip!/Figure 3-Source data 3-part1/1081-1-CRISPResso_Report_8zosk6yj.zip!/CRISPResso_on_1081_1/1a.Read_barplot.pdf]

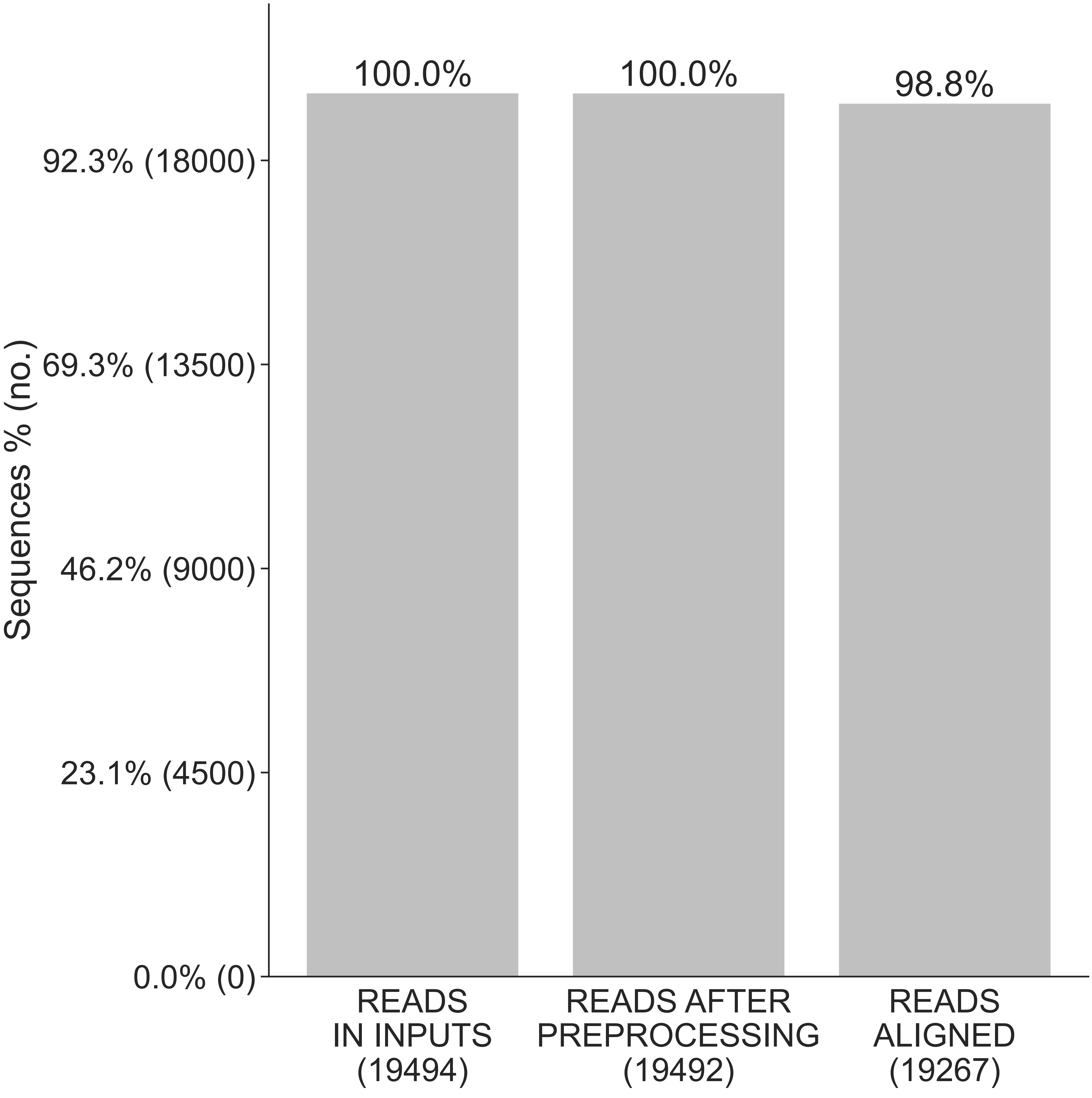
Sequences % (no.)
92.3% (18000)
69.3% (13500)
46.2% (9000)
23.1% (4500)
0.0% (0)
100.0%
100.0%
98.8%
READS IN INPUTS (19494)
READS AFTER PREPROCESSING (19492)
READS ALIGNED (19267)
